# User Experiences With an SMS Text Messaging Program for Smoking Cessation: Qualitative Study

**DOI:** 10.2196/32342

**Published:** 2022-03-18

**Authors:** Alexandra Budenz, Kisha Coa, Emily Grenen, Brian Keefe, Amy Sanders, Kara P Wiseman, Maria Roditis

**Affiliations:** 1 Tobacco Control Research Branch National Cancer Institute Rockville, MD United States; 2 Centers for Medicare and Medicaid Services Woodlawn, MD United States; 3 ICF Fairfax, VA United States; 4 Department of Public Health Sciences University of Virginia Charlottesville, VA United States

**Keywords:** smoking cessation, text messaging interventions, qualitative research, mobile phone

## Abstract

**Background:**

Mobile health strategies for smoking cessation (eg, SMS text messaging–based interventions) have been shown to be effective in helping smokers quit. However, further research is needed to better understand user experiences with these platforms.

**Objective:**

This qualitative study aims to explore the experiences of real-world users of a publicly available smoking cessation program (SmokefreeTXT).

**Methods:**

Semistructured phone interviews were conducted with 36 SmokefreeTXT users between March and July 2014. Of these 36 participants, 50% (18/36) of participants completed the SmokefreeTXT program (ie, did not opt out of the program before the 6- to 8-week completion period), and 50% (18/36) did not complete the program (ie, requested to opt out of the program before the completion period). Interview questions focused on smoking behaviors, quitting history, opinions on the program’s content and structure, answering assessment questions, using keywords, reasons for opting out, and perceived usefulness of the program for quitting smoking. A thematic content analysis was conducted, with a focus on themes to increase program engagement and optimization.

**Results:**

The findings highlighted features of the program that participants found beneficial, as well as some elements that showed opportunities for improvement to boost program retention and successful cessation. Specifically, most participants found the SmokefreeTXT program to be convenient and supportive of cessation; however, some found the messages to be repetitive and reported a desire for more flexibility based on their readiness to quit and cessation progress. We also found that program completion did not necessarily indicate successful smoking cessation and that program opt out, which might be interpreted as a less positive outcome, may occur because of successful cessation. Finally, several participants reported using SmokefreeTXT together with other evidence-based cessation methods or non–evidence-based strategies.

**Conclusions:**

Qualitative interviews with real-world SmokefreeTXT users showed high program acceptability, engagement with program features, and perceived utility for smoking cessation. Our findings directly informed several program updates, such as adding an adaptive quit date feature and offering supplemental information on live support services for users who prefer human interaction during the cessation process. The study has implications for other digital tobacco cessation interventions and highlights important topics that warrant future research, such as the relationship between program engagement (eg, opt out and retention) and successful cessation.

## Introduction

### Background

Although cigarette smoking rates have declined over time, smoking remains the leading preventable cause of death in the United States [1]. In 2019, the prevalence of cigarette smoking among US adults was 13.7% [2]. Although evidence-based approaches, such as smoking cessation counseling and pharmacological cessation aids, can support cessation, they are underused [3,4]. Mobile health (mHealth) smoking cessation platforms (eg, mobile phones, smartphones, and tablets) can be beneficial to the cessation process, as they can reduce barriers (eg, time commitment and access limitations) associated with traditional modalities and have been found to facilitate smoking abstinence [5-9]. mHealth interventions also provide anonymity and real-time support, reaching users from the convenience of their mobile devices at any place or time of day. A type of mHealth intervention with demonstrated efficacy for smoking cessation is an SMS text messaging–based cessation program [5,10,11]. Given the popularity of these programs, pervasive ownership and use of mobile phones in the United States (97% of US adults report owning a mobile phone) [12], and opportunities to reach underserved populations with mHealth smoking cessation programs, it is important to continually optimize these programs.

Despite the demonstrated efficacy of SMS text message–based smoking cessation programs in quantitative evaluations, qualitative studies of user experiences with SMS text message cessation programs are scarce [13-15]. The few qualitative studies that exist on this topic have found high levels of acceptability toward SMS text message–based cessation programs, and participants have reported that they appreciate the convenience and emotional support that these programs offer. However, these studies have primarily been conducted as part of existing cessation trials or with specific populations such as women with pregnancies [13-16]. Moreover, prior studies have been conducted with participants who remained in the cessation SMS text messaging program under study for the entirety of the program, and the experiences of users who opted out of the program before completion were not investigated [13-15].

To address these gaps, we explore the experiences of real-world users of a publicly available smoking cessation program (SmokefreeTXT). Specifically, we conducted qualitative interviews with real-world users (ie, who were not part of an existing research study) of the National Cancer Institute’s (NCI) publicly available SmokefreeTXT program to understand perceptions about program structure and content, engagement with specific program features, and perceived utility of the program for smoking cessation. We also investigate potential differences in program experience between users who completed the SmokefreeTXT program and those who opted out of the program before completion.

### SmokefreeTXT Program

SmokefreeTXT is a free, publicly available, fully automated SMS text message–based smoking cessation program introduced in 2011 by the NCI [8]. The program was developed by a team of mobile technology specialists and clinical psychologists with expertise in tobacco cessation. In 2020, a total of 32,633 new users were enrolled in the SmokefreeTXT program designed for general adult smokers [7].

SmokefreeTXT comprises 6 to 8 weeks of SMS text messages that provide cessation motivation, tips on preparing to quit, advice on managing cravings, quit smoking facts, and recognition of cessation milestones. SmokefreeTXT also refers program users to Smokefree web resources for more detailed smoking cessation information, as well as to the National Network of Tobacco Cessation Quitlines and the NCI Cancer Information Service. Individuals can enroll in SmokefreeTXT through the Smokefree website or by texting a keyword to a short code (QUIT to 47848). At the time of enrollment, users are prompted to set a quit date within 2 weeks of enrollment. Depending on when users set their quit date, they can receive up to 2 weeks of preparation messages leading up to that date. Starting on the quit date, the 6-week intervention comprises 1 to 5 SMS text messages each day, including behavioral intervention and social support messages that target smoking cessation goals [7]. Messages also contain links to relevant pages on the Smokefree website and promote social support through Smokefree social media pages (eg, Facebook).

Throughout the program, users receive messages that assess their smoking status (eg, “Did you smoke today? Reply YES or NO”*)*, craving level (eg, “What’s your craving level? Reply with: HI, MED, or LOW”), and mood status (eg, “How is your mood? Reply GOOD, OK, or BAD”). Users can receive additional messages on demand by texting specific keywords that signify their needs (eg, *MOOD* if they are experiencing a negative mood, *CRAVE* if they have a craving, or *SLIP* if they have smoked a cigarette). Users may also opt out of the program at any time by texting the word *STOP* or reset their quit date at any time by texting the word *NEW* [7]. Completion of the program was defined as not opting out of the program before the conclusion of the 6- to 8-week intervention. See Multimedia Appendix 1 for examples of the program messages.

## Methods

### Recruitment and Participants

Between January and June 2014, all users who completed the SmokefreeTXT program or requested to opt out of the SmokefreeTXT program before program completion were eligible for the study. Program completers (defined as users enrolled in SmokefreeTXT for the duration of the 6- to 8-week program—depending on when they set their quit date—and not opting out before program completion) and noncompleters (defined as users who texted the word *STOP* to opt out of SmokefreeTXT before program completion) were sent an SMS text message inviting them to participate in a phone interview about the program. Interested participants were asked to complete a web-based screener that inquired about their age, race or ethnicity, sex, education, and location (state). Of the 285 individuals who completed the screener, 84.2% (240/285) were eligible to participate in the study (45/285, 15.8% were ineligible because of being aged <18 years or not including contact information on the screener). Eligible respondents were contacted to schedule interviews. We purposively sampled an equal number of men and women as there are documented gender differences in smoking cessation [17]. We also purposefully sampled an equal number of completers and noncompleters, as prior studies of SmokefreeTXT showed differences in experiences with the program based on completion status [8,18]. The first 36 participants who met the recruitment goals (ie, half were men and half were women; half were program completers and half program were noncompleters) and were available to participate in the study were selected. The sample size was determined a priori because of timeline and funding logistics, widely cited literature on qualitative sample size [19-21], and project goals of maximizing variety and depth of the findings within each subgroup while also ensuring that the number of participants included met our purposive sampling criteria (equal numbers by gender and completion status).

Interviews were conducted by 2 experienced research associates with training in behavioral science and extensive experience conducting qualitative research. One of the researchers (Sondra Dietz) held a master’s in public health, and the other (Bethany Tennant) held a doctorate in health education and behavior. Before the interview, participants were informed that the objective of the interview was to gather their feedback on SmokefreeTXT and that interviewers worked for ICF International Inc, a management consulting firm supporting the NCI. The interview questions focused on smoking behaviors, quitting history, opinions on the program’s content and structure, answering assessment questions, using keywords (eg, texting CRAVE to receive additional messages related to smoking cravings), reasons for opting out, and perceived usefulness of the program for quitting smoking. The interview guide was reviewed by subject matter experts in mHealth interventions and smoking cessation but was not pilot-tested with adults who smoked. See Multimedia Appendix 2 for the interview guide. Most interviews lasted 30 to 40 minutes, and all transcripts were recorded and transcribed. Only the participants and members of the research team (ie, an interviewer and a notetaker [Sondra Dietz and Bethany Tennant]) were present on the calls. The interviewers read a consent script by phone and obtained verbal consent before the interview. Participants were compensated for their participation with a US $25 electronic gift card. After the compensation, participants were not contacted again (eg, to review transcripts or study findings).

### Ethical Considerations

The study was approved by ICF's institutional review board (IRB), which holds Federalwide Assurance (FWA 00002349) from the HHS Office for Human Research Protections.

### Data Analysis

The transcripts were analyzed using thematic analysis, an approach in which themes are extracted from qualitative data by identifying salient portions of the transcripts, codes are applied to salient text portions, and codes are extrapolated into larger themes according to their relationship with the study topics of interest [22]. This process mainly relied on deductive coding [23], which was informed by themes that could potentially guide program optimization, such as feedback on program structure and content, but also allowed flexibility for additional codes that emerged. The data analysis was conducted by 3 ICF staff members and 1 NCI staff member, all of whom were trained in either health behavior, public health, or both, and were experienced qualitative researchers. To develop the initial codes, 4 transcripts were repeatedly reviewed, notated with analytic memos throughout the coding process, and analyzed to devise a list of codes. The coders then met to discuss the codes and any discrepancies to reach a consensus, after which point, a preliminary codebook was formed [24]. Next, 2 transcripts were coded using the preliminary codebook. The codebook was further refined based on discussions between the coders to clarify definitions and remove duplicative codes. Using the final codebook, all 36 transcripts were coded in NVivo (QSR International). This process was iterative, and if new codes emerged during the full analysis, the coders convened to discuss the code, gain consensus, and update the codebook and previously coded transcripts accordingly. After all transcripts were coded, groups of codes were categorized and interpreted as larger themes [23]. Themes were further refined through a rereview of the transcripts using negative case analysis, wherein the coders discussed findings from *outlier* participants whose reports conflicted with most of the sample [25]. For example, most participants reported opting out of the SmokefreeTXT program as they began smoking again or successfully quit smoking; however, a small number of participants reported opting out for other reasons (eg, technology issues), causing the initial hypothesis that opt out was driven only by smoking status to be reconsidered. Although coders looked for differences in responses between men and women and completers and noncompleters, no substantive differences were found. Thus, results are reported for all participants combined, except for the results from questions only asked to noncompleters.

## Results

### Overview

A total of 36 adults, of whom 18 (50%) had completed the program (*completers*) and 18 (50%) had not (*noncompleters*), participated in semistructured, in-depth, individual telephone interviews conducted between March 2014 and July 2014. Most participants had some college education or higher (24/36, 67%), were White (29/36, 81%), and were from the Southern or Northeastern United States (24/36, 67%). The participants’ average age was 36 years (SD 13.5; range 18-60 years; Table 1).

At the time of the interview, participants were asked about their cigarette smoking status, and 53% (19/36) of participants reported that they were no longer smoking (13/18, 72% of completers and 6/18, 33% of noncompleters), whereas 47% (17/36) reported that they were current smokers (5/18, 28% completers, and 12/18, 67% noncompleters). Half of the noncompleters who reported smoking at the time of the interview (6/12, 50%) smoked a pack of cigarettes or more per day, whereas only one of the completers who reported smoking at the time of the interview smoked a pack or more per day. Some participants also reported the use of other tobacco products (eg, cigars, e-cigarettes, hookah, and smokeless tobacco) during the interviews. Approximately 31% (11/36) of participants reported current use of other tobacco products at the time of the interview (3/18, 17% completers and 8/18, 44% noncompleters). See Table 1 for tobacco use behaviors in the sample.

Several primary themes emerged during the thematic analysis of participant interviews. Participants discussed their experiences with the SmokefreeTXT program and perceptions of message content, timing, and frequency. Participants also discussed their use of program engagement features, such as on-demand keywords (eg, *CRAVE*) and assessment questions (eg, *How is your mood today?*). Program noncompleters discussed the reasons for opting out of the program before program completion. Participants also discussed the role of SmokefreeTXT in their quitting process and reported the concurrent use of other cessation strategies with SmokefreeTXT.

**Table 1:** SmokefreeTXT user participants’ demographics and tobacco use behaviors (N=36)^a^.

Characteristics			Total sample		Completers (n=18)		Noncompleters (n=18)
**Gender, n (%)**							
	Male	18 (50)		9 (50)		9 (50)	
	Female	18 (50)		9 (50)		9 (50)	
Age (years), mean (SD; range)			35.9 (13.5; 18-60)		39.3 (12.4; 19-60)		32.5 (13.7; 18-55)
**Race or ethnicity, n (%)**							
	White	29 (81)		14 (78)		15 (83)	
	Black	5 (14)		3 (17)		2 (11)	
	Hispanic	1 (3)		0 (0)		1 (6)	
	Asian	1 (3)		1 (6)		0 (0)	
**Education, n (%)**							
	Less than high school	1 (3)		0 (0)		1 (6)	
	High school or equivalent	11 (31)		4 (22)		7 (39)	
	Some college	12 (33)		8 (44)		4 (22)	
	Graduated college	10 (28)		4 (22)		6 (33)	
	Postcollege education	2 (6)		2 (11)		0 (0)	
**Geographic region, n (%)**							
	Northeast	10 (28)		7 (39)		5 (28)	
	South	14 (39)		7 (39)		5 (28)	
	Midwest	6 (17)		3 (17)		4 (22)	
	West	6 (17)		1 (6)		4 (22)	
**Smoking status at time of interview^b^, n (%)**							
	Smoker	17 (47)		5 (28)		12 (67)	
	Ex-smoker^c^	19 (53)		13 (72)		6 (33)	
**Smoking heaviness at time of interview^d^(n=17), n (%)**							
	<1 (pack per day)	10 (59)		4 (80)		6 (50)	
	≥1 (pack per day)	7 (41)		1 (20)		6 (50)	
**Other tobacco use at time of interview, n (%)**							
	Other tobacco user	11 (31)		3 (17)		8 (44)	
	Not other tobacco user	25 (69)		15 (83)		10 (56)	

^a^Owing to rounding, some percentages may not add up to 100%.

^b^Asked of each participant during the interview.

^c^Defined as participants who reported that they did not smoke cigarettes; may include people who reported using other tobacco products.

^d^Asked of each participant who reported smoking at the time of the interview (n=17).

### Participant Experiences With SmokefreeTXT

#### Convenience of a Mobile-Based Medium

Participants responded positively to the mobile phone–based delivery of SmokefreeTXT, noting its convenience. Some reported that they appreciated the SMS text messaging format as they had already spent a great deal of time on their phones:

I like the whole idea because I’m always on my phone and so, you know, consistent text messages were, you know, great reminders of the fact that I am quitting and that I want to quit and I need to quit. So, I liked it.

program completer, female, cigar smoker

Others similarly noted they could quickly read a text, which was preferable to engaging with more time-consuming cessation resources:

I like the text messages because it pops up during the day, and the job that I do, that works out better, because I check my text messages every so often. And I can’t never answer the phone, and I don’t have time to search the web. But I’ve got time to get a really quick text.

program noncompleter, female, smoker

#### Accountability and Social Support

Several participants reported that they liked having the program *check up* on them, especially when messages aligned with cravings:

...It’s just an excellent reminder for me, especially if I happen to be thinking about doing that [smoking]...all of a sudden...I get this text...it just was an excellent reminder.

program completer, male, smoker

Approximately half of the participants said they felt that SmokefreeTXT was supportive, and one of the participants said it was “like my pocket buddy” (program completer, male, ex-smoker). Some reported that they felt especially supported by SmokefreeTXT as they lacked social support from family and friends or were hesitant to share their quit attempts with others, as they felt judged by previous failed quit attempts:

...None of my friends like here are like smokers, so I can’t tell them like “oh my god, I’m craving a cigarette so bad right now”...I guess this might sound weird, but like I could text someone who like understands...

program completer, female, ex-smoker

#### Automation and Human Interaction

Many participants reported that they disliked that the program sent automated messages and did not include human interaction, reporting that the messages were repetitive, *too automated*, and like *cookie-cutter lines*:

Sometimes, I’d get the same message, and it wouldn’t be the same messages in a row, but it seemed like sometimes, it was pretty generic...It’d be the same sort of three messages.

program noncompleter, male, ex-smoker

A participant reported strongly disliking the program, explaining that he did not feel that an automated program that did not directly provide live support gave enough assistance to promote successful cessation:

Because it was talking to a machine...Some of it was just repetitive. There was no real emotional support there at all.

program completer, male, smoker

#### Message Tone and Content

The participants had varying perspectives on the preferred message tone. Approximately half of the participants reported that they preferred the positive tone of the program, with messages focusing on the benefits of quitting rather than the negative aspects of smoking. Participants reported that a supportive tone was particularly beneficial when they were facing struggles during their quit attempts:

I liked that, like if you did slip, it wasn’t like oh, my God, how dare you. It would be like...do you want to start over again? Because like that’s happened to me before. It was good.

program completer, female, ex-smoker

However, some participants wanted a *tougher* tone to encourage accountability:

Give me the more serious stuff. Don’t tell me it’s okay to have a slip, because honey, I’ll have slips all day then...I need some really butt-kicking stuff.

program completer, female, smoker

Some participants reportedly preferred a mix of message tones depending on their mood or how confident they felt about their quit attempt:

I think it would be half and half. If you leave work, you might need a more supportive message if you had a bad day.

program noncompleter, male, ex-smoker

Finally, some participants reported that the content and tone of the messages may have been an impediment to cessation. A few said that the messages triggered smoking cravings, and some noted that certain messages seemed to *permit* smoking by framing quit attempt failures positively:

...One that said, even if you fail, if you smoke, don’t give up...like it was ok...I slipped once. I was like, no, don’t tell me that it’s ok to slip once.

program noncompleter, female, ex-smoker

#### Message Frequency, Timing, and Duration

Most participants reported that the number of daily messages received in the program was appropriate; however, some mentioned that they would have preferred either more or fewer messages per day. In addition, several participants suggested that the timing of messages could align better with their personal schedules or typical smoking times:

If you could set the timing, that would be a plus...I tend to smoke early in the morning and late at night...if you can set the frequency on when you do it, that would be an excellent plus because you’re going to get that reminder at the times that you actually may be smoking.

program completer, male, smoker

Some participants also wanted to be able to customize message frequency or program duration so that the program could be abbreviated if the user successfully quit or extended if the user was struggling to quit:

...I wanted more messages...because I wasn’t doing so good in any of my quits. Whereas, like this time next week, you know, maybe I am...doing awesome and it will be the perfect timing...Like maybe there could be some type of...where it says “crave” or “doing good”...just maybe “having a rough time”...and that’s a trigger to the system to say...“extend her out for three more weeks.”

program completer, female, smoker

A few participants noted that having an adaptive quit date (eg, 1 in the past and 1 that could be postponed) would make the program better suited to fluctuating levels of readiness to quit.

### Program Engagement Features

#### Program Keywords

Most participants noted that they liked using the keyword feature when they were struggling with their quit attempt:

It responds quickly when you text “crave.” I like the instant response when you tell it that you are having a problem. It gives me ideas of how to stay strong.

program noncompleter, female, smoker

However, some participants did not use the keywords as they did not remember that this feature was available to them.

#### Assessment Questions

Most participants reported that they liked receiving and responding to the assessment questions. Participants reported a variety of reasons for liking assessments, including their interactivity, accountability, and utility for smoking trigger recognition:

...if I would get the [assessment] message during a particular time of day, it would help then...I would stop and think about the things that were going on around me at that time, and then based on what I replied to the message and what my craving level was...I could say...these are the things that are making me crave.

program completer, male, ex-smoker

However, many participants reported barriers in responding to assessments, including not having time to respond, not recalling the messages, or feeling guilty for having smoked or relapsed:

I’m smoking, and yet, it thinks that I’ve been quit for five days. Don’t tell. So, I’m not responding to it, but that’s because I know I slipped up. But at the same time, I still want the messages to come through.

program completer, female, smoker

### Program Opt Out (Noncompletion)

#### Smoking Status and Opt Out

Among program noncompleters, more than half reported opting out because they were not successful in quitting smoking. A few participants expressed guilt about continuing to use the program while they were still smoking:

Why did I opt out? Well, I started smoking again...It just would make me feel like I was lying, you know? Even if I typed in “slip,” when I throw back a pack and a half a day, that’s not a slip. That’s a habit again.

program noncompleter, male, smoker

Some noncompleters reported opting out as they had successfully quit smoking, and one of these participants reported opting out as the messages were becoming a trigger to smoke after he had quit.

#### Program Factors Influencing Opt Out

Some noncompleters reported that they opted out as a result of the volume and content of the SMS text messages. Some reported that the number of texts received per day contributed to opt out, either reporting that the number of texts per day was too high or that the decrease in messages over time was a factor in opting out:

They kind of slowed down the texts, and...it kind of made me feel that I didn’t need it anymore...The messages weren’t as strong.

program noncompleter, female, ex-smoker

Some noncompleters also reported that they opted out because the messages on their own were not enough of a motivator to quit:

I’d read the text...and then soon as I would close it out light a cigarette within two minutes...I don’t think it did a trigger...It just didn’t do anything to prevent it...it didn’t really help me to say, “okay, I’m not going to.”

program noncompleter, male, smoker

#### Other Factors Influencing Opt Out

Along with smoking status and SMS text message–related factors that contributed to opt out, a few participants reported that they had technical issues with their mobile phones that contributed to opt out. One of the participants reported that he no longer needed the program because he had enough social support at the time.

### The Role of SmokefreeTXT in the Quitting Process

Several participants reported that they were still smoking at the time they enrolled in SmokefreeTXT, whereas fewer said they had started their quit attempt before enrollment. Several participants reported that they joined SmokefreeTXT during one of the multiple quit attempts, and some reported that they were enrolled in the program multiple times, re-enrolling in the program after completion or opt out:

...I was like, you know, I really want to quit. So I thought like maybe this [SmokefreeTXT] will work. And I tried it the first time and I quit for a while and...I had to restart it because I started smoking again. And then I was like I quit for a while and then like things just got crazy and I started smoking and then I was like “I’m going to text the [SmokefreeTXT] number again and it’s going to be the last time I do it.”

program completer, female, ex-smoker

Approximately half of the participants reported that they felt that SmokefreeTXT had a positive impact on their quitting process, calling the program *helpful* and reporting that it made them feel more confident about quitting. Several participants who were smoking at the time of the study reported that, although they did not fully quit while enrolled in the program, the program helped them quit temporarily or reduce their smoking (fewer cigarettes per day and smoking less frequently).

### Other Cessation Support in Conjunction With SmokefreeTXT

Half of the participants reported that although SmokefreeTXT was a useful component of their quit plan, they also relied on other strategies. Notably, several participants reported using strategies that were not evidence-based, such as switching to smokeless tobacco or using hypnosis rather than evidence-based methods such as over-the-counter nicotine replacement therapy (NRT) or prescription cessation medications. Although there are no reports on the implications for SmokefreeTXT on the effectiveness of or adherence to NRT, a couple of participants reported they were motivated to use NRT while they were enrolled in the SmokefreeTXT program, and a few participants reported that they would like SmokefreeTXT to facilitate access to NRT, such as by providing coupons. Several participants also reported using other mHealth cessation resources while enrolled in SmokefreeTXT, such as cessation smartphone apps and social media focused on cessation support (eg, Smokefree Women Facebook page and web-based smoking cessation forum). Some participants who reported using cessation smartphone apps reported that they would like features of cessation apps to be incorporated into SmokefreeTXT, including tracking money saved by not smoking and smoking milestones (number of days since quitting).

## Discussion

### Principal Findings

Interviews with SmokefreeTXT users showed high program acceptability, engagement with program features, and perceived utility for smoking cessation. Participants provided feedback on program content and functionality, which informed changes to the program. Moreover, the findings provide insights into the complex relationship between program engagement and cessation, which warrants future research.

This qualitative study adds to the limited body of literature that assesses user experiences of smoking cessation SMS text messaging programs. A unique component of this study is its focus on the experiences and perceptions of real-world users of the publicly available SmokefreeTXT smoking cessation SMS text messaging program, which, to our knowledge, has not been explored by other studies of cessation SMS text messaging programs [13-16]. The findings highlighted features of the program that participants found beneficial, as well as some elements that showed opportunities for improvement to boost program retention and successful cessation. We also found that program completion does not necessarily indicate successful smoking cessation and that program opt out, which may be interpreted as a less positive outcome [18], may occur because of successful cessation. Finally, several participants reported using SmokefreeTXT together with other evidence-based cessation methods or non–evidence-based strategies.

As has been found in prior qualitative studies, participants reported that SmokefreeTXT was easier to use than other live support services that require a more significant time commitment [13,14]. They also noted that the anonymity of the platform provided a source of discrete support without perceived judgment or stigma from family or friends [14]. However, similar to what has been found previously, several participants disliked the automated nature of the program, the absence of human interaction, and what they perceived as generic-sounding messages [14,15].

In terms of program structure, many participants reported that they would like the ability to customize message timing, program duration, and quit date settings, which has been reported in previous studies [13,16]. This underscores the need for mobile-based programs to take into consideration that cessation is a highly individualized and often nonlinear process, which may be optimally supported by programs offering flexible structure and functionality.

Although participants liked that the keyword feature allowed them to receive on-demand support and that the smoking assessment questions provided accountability, in some instances, participants reported that assessments triggered feelings of guilt if they had relapsed, which mirrors previous research [13]. In addition, some participants felt that they could not respond accurately to assessment questions (eg, a question about *slips* may not feel relevant if a user had relapsed). Therefore, as quitting smoking is rarely a linear process, it is important to consider other metrics of program success in addition to abstinence [26]. For example, some participants in our study reported that the SmokefreeTXT program helped them quit temporarily, and participants commonly reported making multiple quit attempts, both of which have been defined as metrics of success in other cessation SMS text messaging studies [13,15]. Participants also noted a reduction in their smoking. Given that many study participants entered the program during one of several quit attempts and that several participants enrolled in the program multiple times, further research is needed to better understand how to meet the needs of smokers who may engage in several quit attempts before being successful. For users who are not yet ready or able to quit, examining intermediate outcomes (potentially through responses to assessment questions), such as the number of quit attempts or cutting back on smoking, may provide valuable information to best support users during different stages of the cessation process.

To our knowledge, this is one of the first qualitative studies of cessation text program users that has explored the factors influencing program opt out. Two existing studies compared the experiences of users who either quit smoking or did not quit smoking while using a cessation SMS text messaging program [13,15]. As participants of those studies were required to complete the program as part of their participation in an existing clinical trial, the studies were unable to examine the characteristics and experiences of users who would have otherwise opted out of the program. Most noncompleters reported opting out of SmokefreeTXT because of smoking relapse. However, not all participants who opted out did so as they had resumed smoking. Some opted out as they had successfully quit smoking and felt that they no longer needed the program, which suggests a need to reconceptualize the implications of program opt out [27]. Therefore, although certain factors may make opt out more likely, this qualitative work underscores that opt out is more nuanced than previous research suggests. In other words, opt out may not be definitively used as an indicator of either continued smoking or abstinence; rather, it is best seen as an indication that the user no longer finds it helpful to receive messages from an SMS text messaging program.

These findings also suggest that strategies to re-engage users that opt out should not be a one-size-fits-all approach. Although smoking relapse and cessation were the primary drivers of opt out, a few participants cited the number of messages as a reason for opting out, and others reported opting out as they did not find the messages sufficiently motivating to help them quit smoking. This reinforces the need to consider the message volume and tone when developing an automated program [13,14]. Opt out may have also been influenced by the level of smoking dependence and other tobacco use in the noncompleters group, as noncompleters tended to be heavy smokers, and many were poly-tobacco users.

Finally, although most participants reported that SmokefreeTXT was a useful component of a quit plan, approximately half relied on other cessation strategies as a complement to SmokefreeTXT. Notably, many of the strategies used by study participants were not evidence-based, and some participants reported supplementing their use of SmokefreeTXT by switching from cigarettes to other tobacco products, exposing them to further health risks associated with tobacco use. Therefore, it is important to consider how SMS text message programs can be used to encourage participants to both find evidence-based support (eg, NRT and prescription cessation medication) and work synergistically with these other supports.

### SmokefreeTXT Optimizations

The results of this study have informed several optimizations to the program. For example, SmokefreeTXT has added messages that provide supplemental information on live support services and has optimized the SMS text message library to ensure that SMS text messages are more varied and less repetitive. Since conducting this study, the SmokefreeTXT program has also been changed to allow users to have a more flexible quit date at the start of the program. Users can set a quit date in the past if they begin their quit attempt before starting the SmokefreeTXT program, and users are also sent a message the day before their scheduled quit date and asked to text *ready* if they would like to keep the quit date they set or *not ready* to postpone the start of their quit attempt and set a new date.

Although we conducted qualitative interviews with users of the SmokefreeTXT program, several findings may also be applicable to the development and optimization of other cessation SMS text messaging programs. First, it may be important to allow users to customize the number of SMS text messages that they receive in the program. Cessation SMS text messaging programs may also be used with other cessation strategies; thus, it may be beneficial for SMS text message content to include messages about evidence-based cessation strategies. Relatedly, live support (eg, tobacco quitlines) is a cessation resource that can be recommended within SMS text message programs to benefit users who prefer human interaction as a component of their cessation process. Finally, program retention versus opt out may have limitations in the ability to predict the cessation *success* of users who opt out or remain in the program.

### Limitations

This study had several limitations. First, we conducted in-depth interviews with a small convenience sample of SmokefreeTXT users who were mostly young, White, and had at least some college education. Thus, the results may not be generalizable to a wider population of smokers in the United States. Most current users of SmokefreeTXT are White (11,383/18,510, 61.50%), which demonstrates the need to consider how the program can better reach other demographic groups. In 2016, the SmokefreeTXT program began collecting race and ethnicity data as part of program opt in to track the demographics of the program over time, and future research recruitment efforts may be informed by these data.

SmokefreeTXT users who agreed to participate in the study may have been highly motivated to provide feedback on the program, whether positive or negative, which may have influenced the results. In addition, smoking status was not biochemically verified and relied on the self-reports of the users during the interviews. It is possible that some users stated that they had quit smoking to be more socially acceptable to the interviewer.

This study was conducted in 2014; since then, both mobile phone technology and the SmokefreeTXT program have evolved. However, although there have been changes to message content and quit date flexibility over time, the overarching structure and features of the SmokefreeTXT program (ie, message timing, keywords, and assessment questions) have remained consistent. Furthermore, the results still provide unique data from users who were not part of an intervention trial, and the SmokefreeTXT program continues to be widely used. Thus, the results can still provide actionable information for those developing new or adapting established cessation SMS text messaging interventions. Given the increasing importance of mHealth as an avenue for cessation, data from this study are still relevant today. In addition, some of the findings presented here may have new relevance as improvements to technology enable some of the recommended optimizations, such as the integration of SMS text messages with other digital resources; for example, quitlines or chat applications.

### Conclusions

Qualitative interviews with real-world SmokefreeTXT users showed high program acceptability, engagement with program features, and perceived utility for smoking cessation. Our findings demonstrate the importance of allowing customization to the frequency of SMS text messages within cessation text programs, the limitations of measuring cessation *success* through program opt out versus retention, and the importance of recommending evidence-based cessation resources (including live support via quitlines for users who prefer human interaction during the cessation process) to be used with smoking cessation text programs.

## Appendix

Multimedia Appendix 1 Example SmokefreeTXT program messages.

Multimedia Appendix 2 Example Moderator Guide.

## Abbreviations

mHealth: mobile health
NCI: National Cancer Institute
NRT: nicotine replacement therapy
