# Supplementary material for: User Experiences With an SMS Text Messaging Program for Smoking Cessation: Qualitative Study
Source: JMIR Form Res. 2022 Mar 18;6(3):e32342. doi: 10.2196/32342 (PMC8976256; doi:10.2196/32342)
Supplement: Multimedia Appendix 1 [file formative_v6i3e32342_app1.docx]

## Multimedia Appendix 1. Example SmokefreeTXT Program Messages

**Example SmokefreeTXT Program Messages**

| **Message Type** | | **Description** | **Example** |
| --- | --- | --- | --- |
| Preparation Messages | | If the user sets a quit date in the future, then they receive up to two weeks of preparation messages leading up to their quit date. | *“SFTXT: One week until quit day! Think about your smoking triggers this week. Knowing your triggers is an important part of understanding why you smoke.”* |
| Main Program Messages | | Starting on the quit date, users receive one to five text messages each day, that provide cessation motivation, tips on preparing to quit, advice on managing cravings, quit smoking facts, and recognition of cessation milestones. | *“SFTXT: If you need extra motivation or support visit us on Facebook: http://lil.ms/2p2b and Instagram: http://lil.ms/2p2d”* |
|  |  |  | *“SFTXT: Stress and anger can be smoking triggers. Stop and breathe. Take deep breaths in through your nose and out through your mouth.”* |
|  |  |  | *“SFTXT: It's been 2 weeks since your quit day - amazing! Think about what you've done for your health and all the time and money you've saved.”* |
| Smoking Assessment | |  | *“SFTXT: We want to know how you're doing. Have you smoked in the last 7 days? Reply: YES or NO”* |
| Craving Assessment | |  | *“SFTXT: Keep your mouth busy with mints, straws, or gum to help to curb cravings. What's your craving level right now? Reply with: HIGH, MED, or LOW”* |
| Mood Assessment | |  | *“SFTXT: How is your mood today? Let us know. Reply: GOOD, OK, or BAD”* |
| On-demand Keywords | | Users can receive additional messages on-demand by texting specific keywords that signify their needs. | *“SFTXT: Use these keywords 24/7 for on-demand support. Text CRAVE, MOOD, or SLIP.”* |
|  | Crave Keyword | Users receive tips managing cravings by texting the keyword CRAVE. | *“SFTXT: Cravings are not the boss of you! Fight the urge to smoke. Drink cold water, have a strong mint, or use mouthwash. It works! Reply CRAVE for more.”* |
|  | Mood Keyword | Users receive tips managing mood by texting the keyword MOOD. | *“SFTXT: Spend 15 mins of quality time with a pet (yours or a friend's). Being with a pet can reduce stress and lower your heart rate. Reply MOOD for more.”* |
|  | Slip Keyword | Users receive advice on managing slip-ups by texting the keyword SLIP. | *“SFTXT: Many ex-smokers say they tried stopping many times before they finally succeeded. Don't let a slip get you down. Reply SLIP for more.”* |
