# Supplementary material for: User Experiences With an SMS Text Messaging Program for Smoking Cessation: Qualitative Study
Source: JMIR Form Res. 2022 Mar 18;6(3):e32342. doi: 10.2196/32342 (PMC8976256; doi:10.2196/32342)
Supplement: Multimedia Appendix 2 [file formative_v6i3e32342_app2.docx]

## Multimedia Appendix 2: Example Moderator Guide

*Please note that different guides were used for male program completers, male program non-compelters, female program completers, and female program non-completers. The following is an example moderator guide (female program completers), which was used in this research.*

### Discussion of Purpose/Introduction of Topic

Hello, my name is [insert]. I work for ICF International, a research and consulting firm contracted by the National Cancer Institute. Thank you for agreeing to talk with me today. I greatly appreciate your time and participation. This interview should take about 30 minutes. Is this still a good time to talk?

ICF is conducting these interviews with women who have used the SmokefreeTXT program. We are interested in getting your feedback on the program. [Read and obtain verbal consent.]

### Background/Warmup Questions

1. Do you own a smartphone or a phone with email or web access?
2. Are you currently smoking?

[Yes]

- 1. Do you consider yourself to be a light (<10 cigarettes/day), moderate (10–19 cigarettes/day), or heavy (>20 cigarettes/day) smoker?
  2. Do you use e-cigarettes?

[No]

- 1. When was the last time you smoked?
  2. Are you using any other tobacco products? E-cigarettes?

1. [If still smoking] Did you quit smoking or cut back at all during the SmokefreeTXT program?

3.1. For how long did you quit?

3.2. How much did you cut back?

3.3. What made you start smoking again?

4. How old were you when you first started smoking?

5. What made you decide to start smoking?

6. [If still smoking] What are some of the reasons you keep smoking?

[If quit smoking] What were some of the reasons you smoked?

6.1. How does/did smoking help you deal with stress?

6.2. How does/did smoking help you manage your weight?

6.3. What are/were some of your fears or worries if you quit smoking?

6.4. How do/did you think quitting will/would impact your health? Your social life

7. What are some of the reasons you want/wanted to quit smoking?

8. How does/did your family or friends influence your smoking?

8.1. Have you ever felt pressured to quit by family or friends? Who?

8.2. Have you ever felt pressured to keep smoking by family or friends? Who?

9. What are some of the things you have done to try and quit smoking? 9.1. What has helped you? What hasn’t?

10. What made you decide to sign up for SmokefreeTXT?

10.1. What about a text program was appealing compared to other quit-smoking methods? 10.2. How confident were you in your ability to quit smoking before you signed up for the program?

10.3. Did signing up make you feel more confident? Why or why not?

### User Needs

11. What are/were your greatest needs or the biggest challenges you face/faced on a daily basis when it comes/came to quitting smoking?

11.1. [If quit smoking] What are your biggest challenges in staying smokefree?

12. What do you recall about how well the messages you received from SmokefreeTXT addressed your needs or challenges?

12.1. How helpful were the messages?

13. What else do you remember about the messages you received?

13.1. How well did the messages make you feel supported?

13.2. Did the messages ever make you want to smoke more?

13.3. What would have made the messages more helpful?

14. What type of information or tone of message do you respond to best (e.g., negative: facts about the harmful effects of smoking; positive: facts about the benefits of quitting)?

14.1. How would you describe the tone of the information you received from SmokefreeTXT?

15. How often do you recall receiving messages from SmokefreeTXT when you first signed up?

15.1. What would you change about the frequency of the messages?

16. What time of day do you recall receiving messages from SmokefreeTXT when you first signed up?

16.1. What would you change about the timing of the messages you received?

17. How often do you receive messages now?

17.1. Do you recall anything about the most recent message you received?

17.1.1. Did it ask you a question?

17.1.2. [If yes] Did you respond? Why or why not?

18. Tell me about the most recent message you received.

18.1.1. Did the message ask you to respond to a question?

18.1.2. [If yes] Did you respond? Why or why not?

19. What would you change about the messages you receive now?

19.1. Content?

19.2. Frequency?

20. As you may remember, SmokefreeTXT has two features that are designed to make the program more interactive and responsive to your needs. An on-demand feature that enables users of the program to text any of the Smokefree keywords (e.g., “crave,” “mood,” “slip”) and get a message back that corresponds to the keyword. So, if you text “crave,” you will get a tip to help you with a craving. A question/reply feature in which the message asks you a question and prompts you to text back a response, such as your craving status.

Which of these features did you use? [If used, go through for each feature]

20.1. What did you like about this feature?

20.2. What did you dislike?

20.3. How did this feature help you deal with a particular issue, such as a craving or stress?

20.4. What would make this feature better?

[If not used, go through for each feature]

20.5. Can you tell me some of the reasons you didn’t use this feature?

20.6. If the program reminded you about this feature, would you have been willing to use it? Can you tell me a little more about that?

20.7. What could be changed about this feature to make you more likely to use it?

[If not familiar with, go through for each feature]

20.8. Does this seem like a feature you would have been likely to use? Why or why not?

21. Thinking back about the entire SmokefreeTXT program, what did you like most? What did you like least?

21.1. Have you or would you recommend the program?

### Continuing Support

22. Do you remember receiving a message that you completed the SmokefreeTXT program?

22.1. [If yes] What did the message say?

22.2. [If yes] At that time, were you still smoking?

22.3. [If no] What would you have liked the message to say?

23. [If still smoking now or when last message was received] Did you ever consider restarting the SmokefreeTXT program?

23.1. What made you decide not to restart the program?

23.2. Were you aware of how to restart the program?

23.3. Do you think you would feel guilty for needing to go through the program again?

23.4. What would make you more likely to restart the program?

24. [If still smoking] What other topics, besides smoking, would you be interested in receiving messages about from SmokefreeTXT?

24.1. How many messages would you be willing to receive on topics other than smoking?

[If quit smoking] Would you have been interested in receiving messages about topics other than smoking from SmokefreeTXT while you were trying to quit? What about now?

24.2. What other topics, besides smoking, would you have been/be interested in receiving messages about from SmokefreeTXT?

24.3. How many messages would you have been willing to receive on topics other than smoking? What about now?

25. [If still smoking] What other tools or resources could the Smokefree program offer that would help you feel better equipped to quit smoking? Address the challenges you face?

[If quit smoking] What other tools or resources could the Smokefree program have offered that would have helped you feel better equipped to quit smoking? Address the challenges you face?

25.1. What tools or resources could the program offer you now?

### Closing

Thank you again for your time today. Your feedback is extremely valuable in helping to improve the SmokefreeTXT program. As a thank-you for your participation, you will be receiving an Amazon e-gift card in the next few days at the email address we have on file for you [note email address on file]. Is this the best email address to send the e-gift card? Before we end our talk, what other comments or suggestions do you have related to SmokefreeTXT? Thanks again. If you have further feedback at a later time, please feel free to follow up with me via email.
